# Quantification of FAM20A in human milk and identification of calcium metabolism proteins

**DOI:** 10.14814/phy2.15150

**Published:** 2021-12-27

**Authors:** Vaksha Patel, Enriko Klootwijk, Gail Whiting, Detlef Bockenhauer, Keith Siew, Stephen Walsh, Markus Bleich, Nina Himmerkus, Graciana Jaureguiberry, Naomi Issler, Jasminka Godovac‐Zimmermann, Robert Kleta, Jun Wheeler

**Affiliations:** ^1^ Department of Renal Medicine University College London London UK; ^2^ National Institute for Biological Standards and Control, Medicine and Healthcare Products Regulatory Agency Hertfordshire UK; ^3^ Institute of Physiology University of Kiel Kiel Germany

**Keywords:** calcium metabolism, enamel renal syndrome, FAM20A and human milk

## Abstract

**Background:**

FAM20A, a recently discovered protein, is thought to have a fundamental role in inhibiting ectopic calcification. Several studies have demonstrated that variants of *FAM20A* are causative for the rare autosomal recessive disorder, enamel‐renal syndrome (ERS). ERS is characterized by defective mineralization of dental enamel and nephrocalcinosis suggesting that FAM20A is an extracellular matrix protein, dysfunction of which causes calcification of the secretory epithelial tissues. FAM20A is a low‐abundant protein that is difficult to detect in biofluids such as blood, saliva, and urine. Thus, we speculated the abundance of FAM20A to be high in human milk, since the secretory epithelium of lactating mammary tissue is involved in the secretion of highly concentrated calcium. Therefore, the primary aim of this research is to describe the processes/methodology taken to quantify FAM20A in human milk and identify other proteins involved in calcium metabolism.

**Method:**

This study used mass spectrometry‐driven quantitative proteomics: (1) to quantify FAM20A in human milk of three women and (2) to identify proteins associated with calcium regulation by bioinformatic analyses on whole and milk fat globule membrane fractions.

**Results:**

Shotgun MS/MS driven proteomics identified FAM20A in whole milk, and subsequent analysis using targeted proteomics also successfully quantified FAM20A in all samples. Combination of sample preparation, fractionation, and LC‐MS/MS proteomics analysis generated 136 proteins previously undiscovered in human milk; 21 of these appear to be associated with calcium metabolism.

**Conclusion:**

Using mass spectrometry‐driven proteomics, we successfully quantified FAM20A from transitional to mature milk and obtained a list of proteins involved in calcium metabolism. Furthermore, we show the value of using a combination of both shotgun and targeted driven proteomics for the identification of this low abundant protein in human milk.

## Graphical abstract

Variants of FAM20A causes enamel‐renal syndrome which is characterized by defective mineralization of dental enamel and nephrocalcinosis. FAM20A is associated in inhibiting calcification. The article describes the process undertaken to quantify this low abundant protein in human milk and using bioinformatical analysis, have identified potential candidate proteins that could be involved in calcium metabolism and could possibly network with FAM20A.

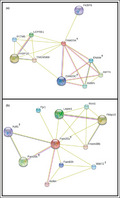

## 1 INTRODUCTION

Human milk is produced by mammary epithelial cells (MEC). The milk produced is comprised of three fractions: the MEC, whey, and milk fat globule membrane (MFGM). All three fractions contain an array of proteins (including immunoglobulins), lipids, carbohydrates, minerals, and bioactive compounds to provide the necessary nutrients and immunity for the developing infant.

Calcium is one of the major minerals that is transported through the MEC during milk production. The amount of calcium ion (Ca^2+^) passing through these MEC during lactation ranges from 200 to 400 mg per day (Kovacs, 2016; Prentice, 2003). During lactation, there is an increase in Ca^2+^ reabsorption via the renal tubular epithelium (Beggs et al., 2017; Ritchie et al., 1998) and in bone demineralization (Kent et al., 1993) with intestinal calcium absorption remaining normal (Kovacs et al., 2000). Furthermore, total serum calcium concentrations are maintained at ~2.4 mM (Kovacs, 2016) and do not differ between lactating, pregnant, or non‐pregnant persons. At physiological serum Ca^2+^ concentrations, nearly half is protein bound (predominantly to albumin) to prevent precipitation. However, the total calcium concentration in human milk is much higher (5–10 mM) (Mahdavi et al., 2015; Perrin et al., 2017; Suzuki et al., 1991) suggesting that calcium buffering would be essential in lactating MEC.

To date, several proteins have been associated with handling Ca^2+^ throughput in the secretory epithelial tissue of the kidney (Hwang et al., 2014; Muto et al., 2010; Zhang & O'Neil, 1996) and breast tissue during lactation (Cross et al., 2013; Reinhardt et al., 2004; Ross et al., 2013; Shennan & Peaker, 2000; Vanhouten & Wysolmerski, 2013). Of particular interest is FAM20A, a member of the Golgi‐Associated Kinase family (Nalbant et al., 2005; Zhang et al., 2018); other members include FAM20B (Koike et al., 2009) and FAM20C (Vogel et al., 2012). FAM20A enhances phosphorylation of extracellular matrix proteins through dimerization with FAM20C (Cui et al., 2015; Tagliabracci, 2015; Tagliabracci et al., 2012). Notably, mutations in *FAM20A* results in enamel‐renal syndrome (ERS) (Jaureguiberry et al., 2012; Kantaputra et al., 2014; Kantaputra et al., 2014; Wang et al., 2013, 2014), a condition characterized by defective mineralization of dental enamel (amelogenesis imperfecta) and calcification within renal tissues (nephrocalcinosis) (Jaureguiberry et al., 2012; Kantaputra, Bongkochwilawan, et al., 2014; Wang et al., 2013, 2014). Histopathological evaluation of FAM20A knockout mice reveal calcification in the renal arteries, the choroid plexus of the brain, the junction between the retina and the ciliary body of the eyes, as well as tissues of the lungs (Vogel et al., 2012). Taken together, these data suggest a significant role for FAM20A in regulating calcium handling within epithelia, and we can assume that FAM20A has a preventive role in ectopic calcification based on associations rather than a direct causative evidence. Furthermore, a recent publication has identified FAM20A protein to be present in lactating but absent in non‐lactating mammary glands (Cui et al., 2015), this suggested a potentially important role for FAM20A in lactation. This is further supported by data from the gene annotation portal, BioGPS (http://biogps.org) which shows mRNA expression of *FAM20A* to be the highest in the lactating mammary gland compared to any other tissues in mouse. Therefore, to investigate this further we aimed to quantify FAM20A in human milk and identify other proteins involved in calcium metabolism.

There have been several shotgun proteomics studies performed mainly on either the whey or MFGM fraction of human milk (Gao et al., 2012; Hettinga et al., 2011; Liao et al., 2011a, 2011b; Palmer et al., 2006; Picariello et al., 2012). Advances in relative quantitative multiplex proteomics techniques of two‐dimensional difference gel electrophoresis (2D‐DIGE) (Molinari et al., 2012; Unlü et al., 1997), tandem mass tags (TMT) (Thompson et al., 2003; Zhang et al., 2013), isobaric tags for relative and absolute quantification (iTRAQ), (Mange et al., 2013; Molinari et al., 2012; Ross et al., 2004; Yang et al., 2015) and dimethyl labeling (Zhang et al., 2016) have improved the ability to obtain a quantitative inventory of differentially expressed proteins across the various stages of milk postpartum. The concentrations of some components change significantly over the three distinct stages of milk postpartum; colostrum (first few days), transitional (up to 3 weeks), and mature (up to 52 weeks plus) milk (Lonnerdal et al., 1987). Previous studies have identified FAM20A (Gao et al., 2012; Mange et al., 2013; Zhang et al., 2013) in human milk but to date, there has been no quantification data on this protein. In biological fluids such as blood, human milk, and CSF where the dynamic range of protein concentration is over 10 orders of magnitude, low abundant proteins are often missed when using mass spectrometry, due to competition with high abundant proteins. Furthermore, where low abundant proteins have been identified through a traditional shotgun proteomic experiment, identification of these low abundant proteins can easily be missed when performing a shotgun quantitative experiment. This is due to the inherent limitation of mass spectrometry. Therefore, this manuscript describes a comprehensive procedure for the identification and quantification of FAM20A from transitional to mature milk and to obtain a list of proteins potentially involved in calcium metabolism.

## 2 METHODS AND MATERIALS

### 2.1 Sample Collection

Human milk samples were donated by three healthy lactating volunteers. The first individual provided milk at 2 weeks, the second at 24 weeks, and the third at 44 weeks postpartum. To assess whether an absence of protease inhibitors (PI) in the samples influenced the identification and quantification of FAM20A, each of the three samples was divided into two aliquots. The first had PI (cOmplete^TM^ Mini EDTA‐free Protease Inhibitor Cocktail, Roche) added to it (one tablet of PI was dissolved in 10 ml of human milk) the second did not. Samples collected at 2 and 44 weeks were processed immediately, and the sample collected at 24 weeks was immediately stored in a domestic freezer prior to it being processed.

**FIGURE 1:**
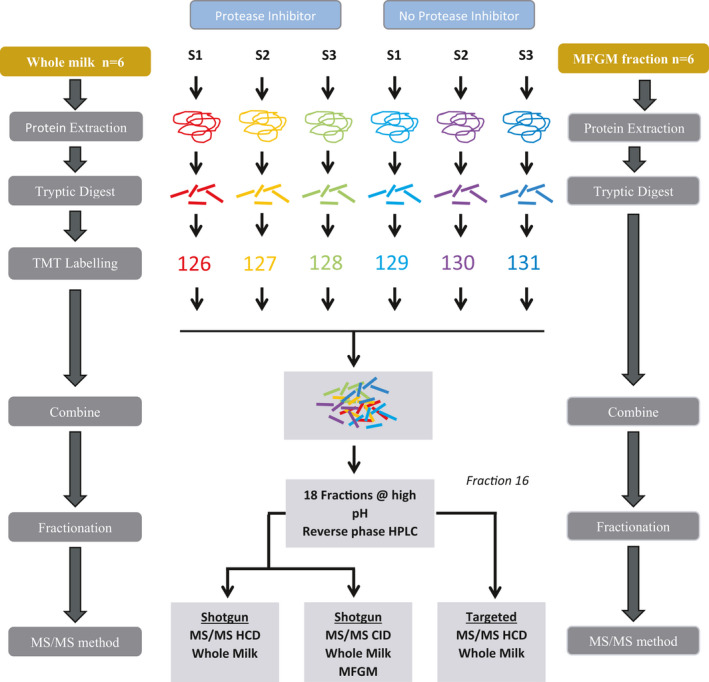
Summary of experimental design and sample processing. Whole milk and MFGM from three human milk samples (sample 1 [S1], sample 2 [S2], and sample 3 [S3]) with and without protease inhibitor were digested with trypsin. Whole milk samples were labeled with TMT sixplex and the MFGM fractions were not labeled. Either the labeled and unlabeled samples were pooled and fractionated by high pH reverse phase chromatography. Proteomic analysis on whole milk was performed using CID MS/MS and HCD MS/MS. Proteomic analysis was MFGM fraction was performed using CID MS/MS

A summary of the experimental design including sample processing is illustrated in Figure 1

### 2.2 Protein extraction

For whole milk, protein extraction was achieved by thoroughly mixing each of the six samples (with and without PI) with equal volumes of 1% (w/v) sodium dodecyl sulfate (SDS). The samples were centrifuged for 10 min at 10,000 *g* at 14°C. Protein estimation was performed on the supernatants using the Bradford protein assay. All samples were diluted to a final total protein concentration of 1 µg/µl in 200 mM triethylammonium bicarbonate. The final percentage of SDS in all samples were below 0.25% (w/v).

Extraction of proteins from the MFGM was performed as described by Fortunato et al. (2003) with slight modification. In short, human milk was centrifuged for 10 min at 10,000 *g*; the fat layers were carefully removed and washed three times with phosphate buffer saline (by centrifugation at 20,000 *g* for 5 min at 14°C) to remove any water‐soluble proteins. The MFGM was then mixed with equal amounts of 1% SDS (w/v) and incubated at room temperature overnight on a rotator. Samples were then centrifuged for 30 min at 20,000 *g* at 14°C. The proteins in each of these solutions were precipitated using acetone, where the final percentage of acetone within each of the sample was 80% (v/v). The samples were left to incubate at −20°C for one hour prior to centrifugation for 10 min at 20,000 *g*. Acetone was removed. Once all the pellets were air‐dried, samples were suspended to the original same volume with water. Protein estimation using the Bradford method was performed on all samples.

### 2.3 Tryptic digestion and TMT labeling

Equal amounts of proteins extracted from human milk of the three control samples (2, 24, and 44 weeks postpartum) with and without PI were taken. The six samples were individually reduced with tris (2‐carboxyethyl) phosphine, alkylated with iodoacetic acid, and precipitated with ice‐cold acetone. The resulting protein pellets were digested with trypsin (Promega). Two and a half micrograms for trypsin was used to digest 100 µg of total protein at 37°C overnight before labeling with either the TMT of 126, 127, 128, 129, 130, or 131 (Thermo Fisher Scientific) according to the manufacturer's instruction and then mixing into a single “sixplex” sample.

Due to low and variable protein concentration in the MFGM samples (0.3–5.4 μg/μl) across sets of samples, tryptic digest was performed on the pooled samples without TMT labeling. The rationale for pooling was to see whether FAM20A could be identified. The amount of protein extracted from the MFGM fraction in one sample was below the optimal conditions (<25 µg) required for TMT labeling. The varying protein content extracted from MFGM samples may be due to the small volume of sample processed and protein extraction efficiency being below optimal conditions.

### 2.4 First dimensional high pH reversed‐phase high‐performance liquid chromatography (RP‐HPLC) separation for peptide mixture

For both the whole milk (TMT labeled) and MFGM (non‐labeled), an aliquot of the samples was subjected to high pH RP‐HPLC separation on a XBridge C18 column (5 µm, 4.6 mm id, and 25 cm long, from Waters) using a linear gradient consisting of mobile phase A (10 mM ammonium formate, pH 10.0) and up to 70% B (90% acetonitrile in A) for 2 h at a flow rate of 0.5 ml/min on a Jasco HPLC system (Jasco). 18 fractions with the retention time of 10–110 min were collected and concatenated.

### 2.5 Nano‐LC and tandem mass spectrometry for protein identification and quantification

The dried high pH RP‐HPLC fractions were resuspended in 0.1% (v/v) formic acid. Online nano‐LC and tandem mass spectrometry (MS/MS) were performed on a LTQ‐Orbitrap mass spectrometer (Thermo Fisher Scientific) coupled with U3000 direct nanosystem (Thermo Fisher Scientific), using a PepMap C18 RP nano column (3 µm, 100 Å, 50 cm long) under a column flow rate of 0.3 µl/min and a gradient of 5%–25% for 180 min, 25%–32% for 20 min, and 32%–90% for 10 min of a solution of 95% (v/v) acetonitrile and 0.1% (v/v) formic acid. In the case of protein identification only, five events of collision‐induced dissociation fragmentation (CID MS/MS) were used for generating spectra for peptide sequencing with data‐dependent acquisition and dynamic exclusion mode enabled. However, in the case of protein identification and quantification, three events of high‐energy collisional dissociation (HCD) were followed by three events of CID to generate spectra both for peptide sequencing and relative quantification via report ions, that is, the TMT tags (HCD MS/MS).

Targeted MS/MS was based on data‐dependent acquisition using the top five most intense peaks and dynamic exclusion enabled. The mass of the peptide of interest was used in the inclusion list so that the mass detector was scanning and looking for the parent ion followed by CID MS/MS.

### 2.6 MS/MS Data analysis

Thermo Proteome Discovery version 1.2 data analysis software was used for mass spectra processing, database searching, and quantification by *in silico* matching of the MS/MS spectra against the UniProtKB human Swiss‐Prot database (https://www.uniprot.org/uniprot/?query=HUMAN&sort=score). Spectra from the 18 fractions were added together as one sample during searching. Initial mass tolerances by MS were set to 10 ppm and up to two missed tryptic cleavages were included. Methionine oxidation was set as dynamic modification whereas carboxymethylation on cysteine and TMTsixplex labels on N‐terminus and lysine side chain were set as static modifications. Protein with two minimal peptides at rank 1 with high confidence was unambiguously identified. Only data generated from the TMTsixplex reporter ion spectra from the peptides that could be uniquely assigned to a given protein were used for relative quantification. TMT reporter ions were detected under HCD mode, and their peak areas were calculated to indicate their intensities.

The percentage abundance was calculated by normalizing the peak area of the individual protein to the sum of peak areas of all proteins identified for each MS/MS experiments.

### 2.7 MS/MS data comparison

The proteins identified from whole milk and MFGM fractions were combined and this list was compared to the entire human milk proteome as mapped out recently by several studies prior to August 2020 (Herwijnen et al., 2016; Lu et al., 2018; Ma et al., 2019; Yang et al., 2017; Zhu et al., 2019). One of the earlier studies by van Herwijnen et al. (2016) aggregated 38 additional studies. The rationale for conducting such a comparison was to investigate whether our alternative approach of a combination of sample preparation and mass spectrometry strategy identified proteins previously undiscovered proteins in human milk and to see whether any of these may be involved in the regulation of calcium.

To determine how the proteins are involved in calcium regulation, a functional classification analysis was performed. This involved extracting GO terms related to relevant keywords (i.e., calcium) with the intent to identify proteins that may be involved in calcium regulation.

Furthermore, since gene names (GN) were used as protein identifiers in the van Herwijnen et al. (2016) publication, all proteins identified in our investigations were therefore converted to their GN for ease of comparison. Any anomalies which included proteins having different accession numbers but sharing the same associated GN, and proteins that do not have a GN assigned, were removed and not included in the final list of comparison.

### 2.8 Bioinformatic analyses

We have chosen two web‐based platforms: STRING 10.5 (Search Tool for the Retrieval of Interacting Genes, http://string‐db.org/cgi/input.pl) and QuickGO (https://www.ebi.ac.uk/QuickGO/) to demonstrate protein‐to‐protein interaction and functional classification, respectively. Both these platforms were selected for their ease of use and having been peer reviewed.

### 2.9 Interaction of FAM20A with other proteins

Network analysis was performed using STRING to identify FAM20A interaction with other proteins. The criteria used were of high confidence interaction score of 0.7, together with interaction evidence based on experiments, co‐occurrence, databases, neighborhood, text mining, fusion, and co‐expression all selected. In addition, not more than 10 interactors were shown for the first shell (direct interaction of proteins to FAM20A).

### 2.10 Functional classification

Functional classification analysis was performed using QuickGO. This software package was used to extract all available GO terms that are relevant to the keyword “calcium.” The software was also used to identify which of the proteins identified in this investigation were associated with the GO term calcium.

QuickGO is a browser‐based application for GO terms and annotations, which contains information for 45919 GO terms, as defined in the version of the GO OWL definition file, accessed on April 20, 2016. (Binns et al., 2009) Only protein with UniProt IDs could be used as input within the QuickGO database.

Proteins within our dataset that are associated with GO calcium term were then extracted using the following settings: molecular function, cellular components, and biological processes enabled, Taxon was set at *Homo sapiens* (9606) enabled, all reference selected, and all databases publicly available at the time of analysis. The output GNs were then matched with the original input list to highlight any anomalies.

## 3 RESULTS

### 3.1 Identification and quantification of FAM20A in whole milk

**FIGURE 2:**
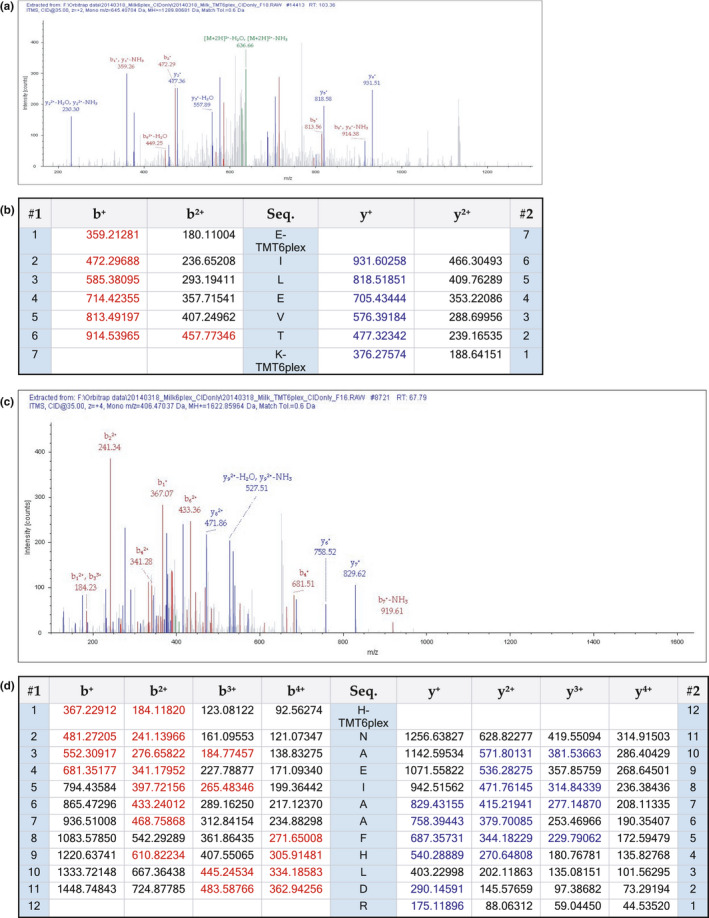
MS/MS spectra of the identified peptides for FAM20A. The (a) EILEVTK (c) HNAEIAAFHLDR peptide spectra were obtained from fractions 18–16 of human milk, respectively. The (b) and (d) depicts the raw masses (red are b ions and blue are y ions) detected and identified EILEVTK and HNAEIAAFHLDR peptide, respectively

In this investigation, three fragmentation experiments were employed. FAM20A was identified in whole milk from fractions 16 and 18 from the shotgun CID MS/MS fragmentation experiment. Identity was based on two peptide fragments of FAM20A; one peptide (HNAEIAAFHLDR) is also found in FAM20B and FAM20C and the other (EILEVTK) was unique to FAM20A (Figure 2). The mass of 645 shown in Figure 2 is the doubly charged species of 1289.086. Ions were selected based on their m/z value, where m = mass and z = charge, and therefore the detected peak is 645 (1289.086/2).

### 3.2 Targeted proteomics: Relative quantification of FAM20A in human milk between the three samples with and without PI

**FIGURE 3:**
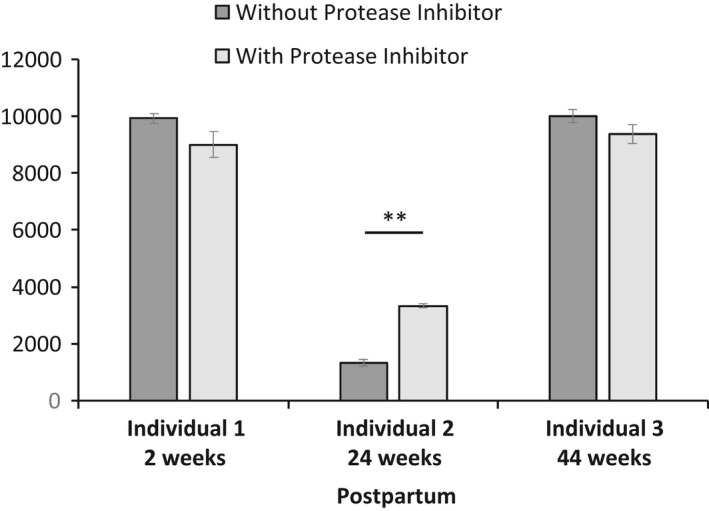
Relative quantification of the unique FAM20A peptide (EILEVTK). The unique peptide, EILEVTK for FAM20A was sequenced 43 times and quantified four times using a targeted method based on a doubly charged *m*/*z* ion, 1289.80681. The reporter ions of the TMTsixplex derived from HCD MS/MS of this peptide were used to obtain relative quantification of EILEVTK four times (*n* = 4; technical replicates) in each of the six samples multiplexed. A Student's *t*‐test was performed on the intensity of the EILEVTK peptide identified in milk with and without PI for each individual. Error bars are mean ± SEM. The *p*‐value is based on a Student's *t*‐test. **p < 0.001

Relative quantification using shotgun HCD MS/MS fragmentation experiment did not identify FAM20A. Therefore, a targeted HCD MS/MS fragmentation experiment was employed. This was achieved by selecting the doubly charged *m/z* ion (1289.80681) from the full MS scan for fragmentation. This doubly charged *m/z* ion was selected on basis of the EILEVTK ion being identified and sequenced using a shotgun CID MS/MS fragmentation experiment. In the targeted approach the peptide was sequenced 43 times and quantified four times in each sample. The TMTsixplex reporter ion spectra generated from this doubly charged ion was used to report the relative abundance of the unique FAM20A peptide (EILEVTK) between the three samples with and without PI (Figure 3).

A comparison was performed for each individual with and without PI using a Student's *t*‐test. Figure 3 shows the mean ± standard error of mean (SEM) of the relative quantification of the reporter ion loss (*n* = 4; technical replicates per measured sample) from EILEVTK.

Little difference in the relative quantification of FAM20A peptide (EILEVTK) was observed when comparing samples with and without PI from each individual sample; further, signal detected from this peptide was similar between the first and third individuals. There was a marked decrease in the observed EILEVTK signal in the second sample when compared to the first and third samples. This second sample had been stored in a domestic freezer prior to processing. Whereas samples from individuals 1 and 3 were processed within 30 min of collection. PI was added to one aliquot prior to sample 2 being thawed. There was a significant (Student's *t*‐test *p* < 0.001) increase in the level of the EILEVTK peptide in sample 2 with PI when compared to the same sample without PI. The total protein content was similar in sample 2 with (5.2 µg/µl) and without (4.5 µg/µl) PI.

### 3.3 FAM20A abundance in human milk

To determine the relative abundance of FAM20A in the pooled sample of whole milk, a plot based on the relative abundance and concentration of the four major milk proteins, serum albumin, lysozyme, lactotransferrin, and lactoalbumin as determined by Fast Protein Liquid Chromatography (Hambraeus et al., 1978) was generated. The relative abundance of these proteins was calculated and expressed as a percentage of the total protein content of milk, assuming the total protein content of milk was 1.1 g/100 ml (Lynch et al., 2004). Since there would always be a linear relationship between the relative abundance (expressed as a percentage) of a protein (be it highly abundant or low abundant) and the total protein concentration, we compared our dataset with Hambraeus et al. (1978).

**FIGURE 4:**
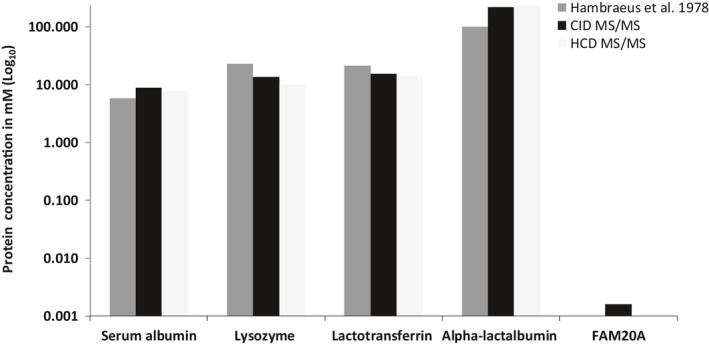
Relative abundance of FAM20A in human milk. Concentration of FAM20A was extrapolated by comparing the relative abundance of major proteins: serum albumin, lysozyme, lactotransferrin, and alpha‐lactalbumin in our human milk sample from both the CID MS/MS and HCD MS/MS dataset with that of the known concentration of the same proteins as determined by Hambraeus et al. (1978). The data shown represents the combined peak area for each protein obtained from the three individual human milk samples with and without PI

The percentage abundance of the stated four major proteins obtained from our CID MS/MS and HCD MS/MS fragmentation experiment was calculated by normalizing the peak area of the individual protein to the sum of peak areas of all proteins identified for each MS/MS fragmentation experiments. However, one limitation of this approach is the under‐representation of the whole milk proteome, therefore the percentage abundance for any given protein identified maybe slightly over‐represented. Nevertheless, the results show that the concentrations of these four major proteins from both our MS/MS datasets were similar to that determined by Hambraeus et al. (1978). Given mass spectrometry is not inherently quantitative because of differences in the ionization efficiency, we were able to perform a comparative analysis on our shotgun CID MS/MS fragmentation experiment to extrapolate and speculate the calculated concentration of 1.6 µM using it as a relative quantification. (Figure 4).

### 3.4 Proteomics profiling and identification of calcium metabolism proteins in human milk

**FIGURE 5:**
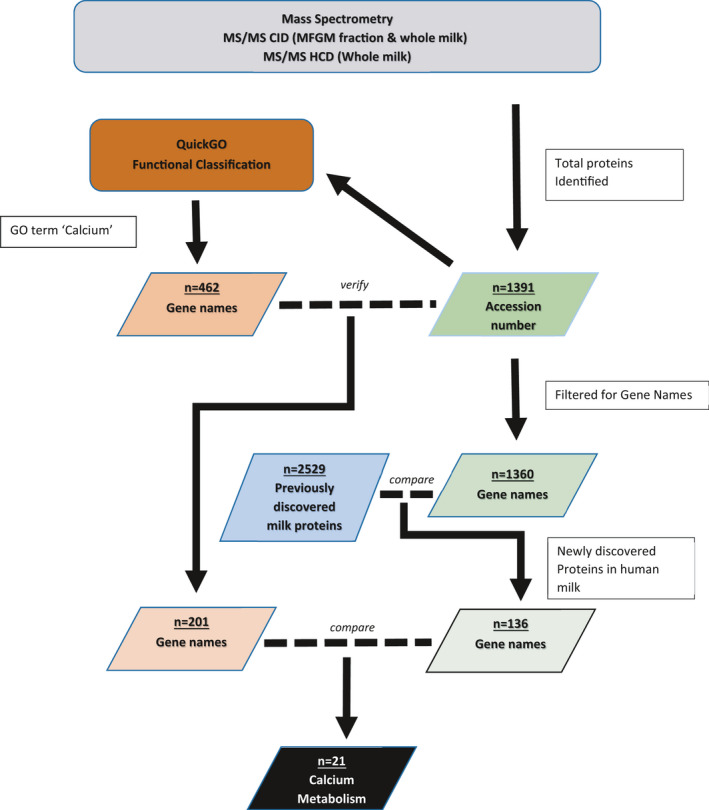
Flow chart on the data generated and how the data were processed. The output data from the fragmentation experiments (CID MS/MS and HCD MS/MS) for whole milk and MFGM fractions were combined and a total of 1391 proteins having accession numbers were identified. The gene names for these 1391 proteins were extracted. Thirty‐one proteins were removed for having either duplicate gene names or no gene names. Comparison on the remaining 1360 proteins with the 2529 previous milk proteome identified 136 previously undiscovered proteins in human milk. The 1391 proteins with accession numbers were analyzed in the QuickGO application to determine which proteins are associated with calcium GO term, the resulting output of 462 proteins was given as gene names. Comparison of these 462 gene names with the gene names extracted from the original input list (1391) gave only 201 matches. Final comparison between these 201 proteins and the list of 136 previously undiscovered human milk proteins identified 21 calcium metabolism proteins that have never been identified in human milk

Figure 5 illustrates the workflow on the data generated for proteomic profiling and the processes involved in the identification of proteins involved in calcium metabolism in human milk.

Shotgun proteomics identified a total of 1391 proteins from both whole milk and MFGM datasets using CID MS/MS and HCD MS/MS fragmentation strategy Data File S1).

In the final list of comparisons, 31 proteins were excluded on the basis of having the same GN but different accession numbers, or if they were without an assigned GN. Subsequently, the remaining 1360 GN were then compared to a previous publication (Herwijnen et al., 2016; Lu et al., 2018; Ma et al., 2019; Yang et al., 2017; Zhu et al., 2019) that lists the entire human milk proteome to date. In fact, this study, which uses an alternative approach of 1% SDS for protein extraction coupled with high pH RP‐HPLC fractionation and nano‐LC‐MS/MS identified 23 and 113 newly discovered proteins in whole milk and MFGM, respectively (Table 1).

**TABLE 1:** Newly discovered proteins in human milk

Milk fraction	MS/MS	Gene name	Accession #	Description	Entry name
MFGM	CID	ABCA13	Q86UQ4	ATP‐binding cassette sub‐family A member 13	[ABCAD_HUMAN]
MFGM	CID	ABCD1	P33897	ATP‐binding cassette sub‐family D member 1	[ABCD1_HUMAN]
MFGM	CID	ACBD5	Q5T8D3	Acyl‐CoA‐binding domain‐containing protein 5	[ACBD5_HUMAN]
**MFGM**	**CID**	**ADGRE2**	**Q9UHX3**	**Adhesion G protein‐coupled receptor E2**	**[AGRE2_HUMAN]**
MFGM	CID	ADGRF1	Q5T601	Adhesion G‐protein coupled receptor F1	[AGRF1_HUMAN]
MFGM	CID	ADGRG2	Q8IZP9	Adhesion G‐protein coupled receptor G2	[AGRG2_HUMAN]
MFGM	CID	AGFG1	P52594	Arf‐GAP domain and FG repeat‐containing protein 1	[AGFG1_HUMAN]
MFGM	CID	ARFGAP1	Q8N6T3	ADP‐ribosylation factor GTPase‐activating protein 1	[ARFG1_HUMAN]
MFGM	CID	ARFGAP2	Q8N6H7	ADP‐ribosylation factor GTPase‐activating protein 2	[ARFG2_HUMAN]
MFGM	CID	ARFGAP3	Q9NP61	ADP‐ribosylation factor GTPase‐activating protein 3	[ARFG3_HUMAN]
MFGM	CID	ATP5I	P56385	ATP synthase subunit e, mitochondrial	[ATP5I_HUMAN]
MFGM	CID	ATP5J	P18859	ATP synthase‐coupling factor 6, mitochondrial	[ATP5J_HUMAN]
MFGM	CID	ATXN2L	Q8WWM7	Ataxin‐2‐like protein	[ATX2L_HUMAN]
MFGM	CID	BCAT2	O15382	Branched‐chain‐amino‐acid aminotransferase, mitochondrial	[BCAT2_HUMAN]
**MFGM**	**CID**	**BLOC1S1**	**P78537**	**Biogenesis of lysosome‐related organelles complex 1 subunit 1**	**[BL1S1_HUMAN]**
**MFGM**	**CID**	**BLOC1S2**	**Q6QNY1**	**Biogenesis of lysosome‐related organelles complex 1 subunit 2**	**[BL1S2_HUMAN]**
**MFGM**	**CID**	**BLOC1S3**	**Q6QNY0**	**Biogenesis of lysosome‐related organelles complex 1 subunit 3**	**[BL1S3_HUMAN]**
MFGM	CID	BLOC1S5	Q8TDH9	Biogenesis of lysosome‐related organelles complex 1 subunit 5	[BL1S5_HUMAN]
MFGM	CID	C19orf25	Q9UFG5	UPF0449 protein C19orf25	[CS025_HUMAN]
MFGM	CID	C1QBP	Q07021	Complement component 1 Q subcomponent‐binding protein, mitochondrial	[C1QBP_HUMAN]
MFGM	CID	C2orf88	Q9BSF0	Small membrane A‐kinase anchor protein	[SMAKA_HUMAN]
MFGM	CID	C4orf32	Q8N8J7	Uncharacterized protein C4orf32	[CD032_HUMAN]
MFGM	CID	CALCB	P10092	Calcitonin gene‐related peptide 2	[CALCB_HUMAN]
MFGM	CID	CASP1	P29466	Caspase‐1	[CASP1_HUMAN]
MFGM	CID	CCDC124	Q96CT7	Coiled‐coil domain‐containing protein 124	[CC124_HUMAN]
MFGM	CID	CCDC134	Q9H6E4	Coiled‐coil domain‐containing protein 134	[CC134_HUMAN]
MFGM	CID	CD320	Q9NPF0	CD320 antigen	[CD320_HUMAN]
MFGM	CID	CDK17	Q00537	Cyclin‐dependent kinase 17	[CDK17_HUMAN]
MFGM	CID	CDNF	Q49AH0	Cerebral dopamine neurotrophic factor	[CDNF_HUMAN]
MFGM	CID	CHCHD3	Q9NX63	MICOS complex subunit MIC19	[MIC19_HUMAN]
MFGM	CID	CNPY3	Q9BT09	Protein canopy homolog 3	[CNPY3_HUMAN]
MFGM	CID	CNPY4	Q8N129	Protein canopy homolog 4	[CNPY4_HUMAN]
MFGM	CID	COX5A	P20674	Cytochrome c oxidase subunit 5A, mitochondrial	[COX5A_HUMAN]
MFGM	CID	COX5B	P10606	Cytochrome c oxidase subunit 5B, mitochondrial	[COX5B_HUMAN]
MFGM	CID	COX6B1	P14854	Cytochrome c oxidase subunit 6B1	[CX6B1_HUMAN]
MFGM	CID	CRADD	P78560	Death domain‐containing protein CRADD	[CRADD_HUMAN]
MFGM	CID	CTAGE5	O15320	cTAGE family member 5	[CTGE5_HUMAN]
MFGM	CID	DDX19A	Q9NUU7	ATP‐dependent RNA helicase DDX19A	[DD19A_HUMAN]
MFGM	CID	DIABLO	Q9NR28	Diablo homolog, mitochondrial	[DBLOH_HUMAN]
MFGM	CID	DNAJC1	Q96KC8	DnaJ homolog subfamily C member 1	[DNJC1_HUMAN]
**MFGM**	**CID**	**DTNBP1**	**Q96EV8**	**Dysbindin**	**[DTBP1_HUMAN]**
MFGM	CID	DYNC1LI1	Q9Y6G9	Cytoplasmic dynein 1 light intermediate chain 1	[DC1L1_HUMAN]
MFGM	CID	DYNLRB1	Q9NP97	Dynein light chain roadblock‐type 1	[DLRB1_HUMAN]
MFGM	CID	EIF2AK2	P19525	Interferon‐induced, double‐stranded RNA‐activated protein kinase	[E2AK2_HUMAN]
MFGM	CID	EIF5B	O60841	Eukaryotic translation initiation factor 5B	[IF2P_HUMAN]
MFGM	CID	EMC1	Q8N766	ER membrane protein complex subunit 1	[EMC1_HUMAN]
**MFGM**	**CID**	**EPS15**	**P42566**	**Epidermal growth factor receptor substrate 15**	**[EPS15_HUMAN]**
**MFGM**	**CID**	**EPS15L1**	**Q9UBC2**	**Epidermal growth factor receptor substrate 15‐like 1**	**[EP15R_HUMAN]**
MFGM	CID	ERICH5	Q6P6B1	Glutamate‐rich protein 5	[ERIC5_HUMAN]
**MFGM**	**CID**	**ERO1A**	**Q96HE7**	**ERO1‐like protein alpha**	**[ERO1A_HUMAN]**
MFGM	CID	F5	P12259	Coagulation factor V	[FA5_HUMAN]
**MFGM**	**CID**	**F7**	**P08709**	**Coagulation factor VII**	**[FA7_HUMAN]**
**MFGM**	**CID**	**F9**	**P00740**	**Coagulation factor IX**	**[FA9_HUMAN]**
MFGM	CID	FAM177A1	Q8N128	Protein FAM177A1	[F177A_HUMAN]
MFGM	CID	FAM213A	Q9BRX8	Redox‐regulatory protein FAM213A	[F213A_HUMAN]
**MFGM**	**CID**	**FKBP7**	**Q9Y680**	**Peptidyl‐prolyl cis‐trans isomerase FKBP7**	**[FKBP7_HUMAN]**
MFGM	CID	GNG10	P50151	Guanine nucleotide‐binding protein G(I)/G(S)/G(O) subunit gamma‐10	[GBG10_HUMAN]
MFGM	CID	GPAT4	Q86UL3	Glycerol‐3‐phosphate acyltransferase 4	[GPAT4_HUMAN]
MFGM	CID	HNRNPDL	O14979	Heterogeneous nuclear ribonucleoprotein D‐like	[HNRDL_HUMAN]
MFGM	CID	HOOK3	Q86VS8	Protein Hook homolog 3	[HOOK3_HUMAN]
MFGM	CID	HSBP1	O75506	Heat shock factor‐binding protein 1	[HSBP1_HUMAN]
**MFGM**	**CID**	**IMMT**	**Q16891**	**MICOS complex subunit MIC60**	**[MIC60_HUMAN]**
MFGM	CID	INPPL1	O15357	Phosphatidylinositol 3,4,5‐trisphosphate 5‐phosphatase 2	[SHIP2_HUMAN]
MFGM	CID	KLC3	Q6P597	Kinesin light chain 3	[KLC3_HUMAN]
MFGM	CID	LAMTOR5	O43504	Regulator complex protein LAMTOR5	[LTOR5_HUMAN]
MFGM	CID	LNP	Q9C0E8	Protein lunapark	[LNP_HUMAN]
MFGM	CID	LRFN1	Q9P244	Leucine‐rich repeat and fibronectin type III domain‐containing protein 1	[LRFN1_HUMAN]
MFGM	CID	MRPL12	P52815	39S ribosomal protein L12, mitochondrial	[RM12_HUMAN]
MFGM	CID	MSLN	Q13421	Mesothelin	[MSLN_HUMAN]
MFGM	CID	MYO18A	Q92614	Unconventional myosin‐XVIIIa	[MY18A_HUMAN]
MFGM	CID	NDUFA5	Q16718	NADH dehydrogenase [ubiquinone] 1 alpha subcomplex subunit 5	[NDUA5_HUMAN]
MFGM	CID	OXR1	Q8N573	Oxidation resistance protein 1	[OXR1_HUMAN]
MFGM	CID	PBXIP1	Q96AQ6	Pre‐B‐cell leukaemia transcription factor‐interacting protein 1	[PBIP1_HUMAN]
MFGM	CID	PCSK1N	Q9UHG2	ProSAAS	[PCSK1_HUMAN]
MFGM	CID	PDRG1	Q9NUG6	p53 and DNA damage‐regulated protein 1	[PDRG1_HUMAN]
MFGM	CID	PEX14	O75381	Peroxisomal membrane protein PEX14	[PEX14_HUMAN]
MFGM	CID	PEX19	P40855	Peroxisomal biogenesis factor 19	[PEX19_HUMAN]
MFGM	CID	PLD2	O14939	Phospholipase D2	[PLD2_HUMAN]
**MFGM**	**CID**	**PRKCA**	**P17252**	**Protein kinase C alpha type**	**[KPCA_HUMAN]**
**MFGM**	**CID**	**PROZ**	**P22891**	**Vitamin K‐dependent protein Z**	**[PROZ_HUMAN]**
MFGM	CID	PXN	P49023	Paxillin	[PAXI_HUMAN]
MFGM	CID	PYM1	Q9BRP8	Partner of Y14 and mago	[PYM1_HUMAN]
MFGM	CID	RAB11FIP1	Q6WKZ4	Rab11 family‐interacting protein 1	[RFIP1_HUMAN]
**MFGM**	**CID**	**RAB3GAP1**	**Q15042**	**Rab3 GTPase‐activating protein catalytic subunit**	**[RB3GP_HUMAN]**
MFGM	CID	RABL6	Q3YEC7	Rab‐like protein 6	[RABL6_HUMAN]
**MFGM**	**CID**	**RCN2**	**Q14257**	**Reticulocalbin‐2**	**[RCN2_HUMAN]**
MFGM	CID	SARG	Q9BW04	Specifically, androgen‐regulated gene protein	[SARG_HUMAN]
MFGM	CID	SCYL1	Q96KG9	N‐terminal kinase‐like protein	[NTKL_HUMAN]
MFGM	CID	SEC63	Q9UGP8	Translocation protein SEC63 homolog	[SEC63_HUMAN]
MFGM	CID	SH3GLB2	Q9NR46	Endophilin‐B2	[SHLB2_HUMAN]
MFGM	CID	SH3YL1	Q96HL8	SH3 domain‐containing YSC84‐like protein 1	[SH3Y1_HUMAN]
MFGM	CID	SHTN1	A0MZ66	Shootin‐1	[SHOT1_HUMAN]
MFGM	CID	SPAG9	O60271	C‐Jun‐amino‐terminal kinase‐interacting protein 4	[JIP4_HUMAN]
MFGM	CID	SRA1	Q9HD15	Steroid receptor RNA activator 1	[SRA1_HUMAN]
MFGM	CID	SRPR	P08240	Signal recognition particle receptor subunit alpha	[SRPR_HUMAN]
MFGM	CID	SSBP1	Q04837	Single‐stranded DNA‐binding protein, mitochondrial	[SSBP_HUMAN]
**MFGM**	**CID**	**STOML2**	**Q9UJZ1**	**Stomatin‐like protein 2, mitochondrial**	**[STML2_HUMAN]**
**MFGM**	**CID**	**SYTL2**	**Q9HCH5**	**Synaptotagmin‐like protein 2**	**[SYTL2_HUMAN]**
MFGM	CID	TACC2	O95359	Transforming acidic coiled‐coil‐containing protein 2	[TACC2_HUMAN]
MFGM	CID	TAPBP	O15533	Tapasin	[TPSN_HUMAN]
MFGM	CID	TFPI	P10646	Tissue factor pathway inhibitor	[TFPI1_HUMAN]
MFGM	CID	TMED7	Q9Y3B3	Transmembrane emp24 domain‐containing protein 7	[TMED7_HUMAN]
MFGM	CID	TMPO	P42167	Lamina‐associated polypeptide 2, isoforms beta/gamma	[LAP2B_HUMAN]
MFGM	CID	TOR1AIP2	Q8NFQ8	Torsin‐1A‐interacting protein 2	[TOIP2_HUMAN]
MFGM	CID	TSC22D4	Q9Y3Q8	TSC22 domain family protein 4	[T22D4_HUMAN]
MFGM	CID	TTC1	Q99614	Tetratricopeptide repeat protein 1	[TTC1_HUMAN]
MFGM	CID	UBAP2L	Q14157	Ubiquitin‐associated protein 2‐like	[UBP2L_HUMAN]
MFGM	CID	UBTD2	Q8WUN7	Ubiquitin domain‐containing protein 2	[UBTD2_HUMAN]
MFGM	CID	UQCRB	P14927	Cytochrome b‐c1 complex subunit 7	[QCR7_HUMAN]
MFGM	CID	UQCRFS1	P47985	Cytochrome b‐c1 complex subunit Rieske, mitochondrial	[UCRI_HUMAN]
**MFGM**	**CID**	**VSNL1**	**P62760**	**Visinin‐like protein 1**	**[VISL1_HUMAN]**
MFGM	CID	ZC3H15	Q8WU90	Zinc finger CCCH domain‐containing protein 15	[ZC3HF_HUMAN]
MFGM	CID	ZNF185	O15231	Zinc finger protein 185	[ZN185_HUMAN]
WM	CID	DDX39B	Q13838	Spliceosome RNA helicase DDX39B	[DX39B_HUMAN]
WM	CID	EEF1A1P5	Q5VTE0	Putative elongation factor 1‐alpha‐like 3	[EF1A3_HUMAN]
WM	CID	EIF2S3L	Q2VIR3	Putative eukaryotic translation initiation factor 2 subunit 3‐like protein	[IF2GL_HUMAN]
WM	CID	H2AFX	P16104	Histone H2AX	[H2AX_HUMAN]
WM	CID	H2BFS	P57053	Histone H2B type F‐S	[H2BFS_HUMAN]
WM	CID	H3F3A	P84243	Histone H3.3	[H33_HUMAN]
WM	CID	HIST1H2AA	Q96QV6	Histone H2A type 1‐A	[H2A1A_HUMAN]
WM	CID	HIST1H2BC	P62807	Histone H2B type 1‐C/E/F/G/I	[H2B1C_HUMAN]
WM	CID	HIST1H2BH	Q93079	Histone H2B type 1‐H	[H2B1H_HUMAN]
WM	CID	HIST1H2BN	Q99877	Histone H2B type 1‐N	[H2B1N_HUMAN]
WM	CID	HIST2H3A	Q71DI3	Histone H3.2	[H32_HUMAN]
WM	CID	NUMA1	Q14980	Nuclear mitotic apparatus protein 1	[NUMA1_HUMAN]
WM	CID	RPL26L1	Q9UNX3	60S ribosomal protein L26‐like 1	[RL26L_HUMAN]
WM	CID	RPL36A	P83881	60S ribosomal protein L36a	[RL36A_HUMAN]
WM	CID	RPL36AL	Q969Q0	60S ribosomal protein L36a‐like	[RL36L_HUMAN]
WM	CID	TKFC	Q3LXA3	Triokinase/FMN cyclase	[TKFC_HUMAN]
WM	CID	UBA52	P62987	Ubiquitin‐60S ribosomal protein L40	[RL40_HUMAN]
WM	CID	UBE2NL	Q5JXB2	Putative ubiquitin‐conjugating enzyme E2 N‐like	[UE2NL_HUMAN]
**WM**	**HCD**	**FYN**	**P06241**	**Tyrosine‐protein kinase Fyn**	**[FYN_HUMAN]**
WM	HCD	HIST1H3A	P68431	Histone H3.1	[H31_HUMAN]
WM	HCD	HIST2H2AB	Q8IUE6	Histone H2A type 2‐B	[H2A2B_HUMAN]
WM	HCD	HSD17B10	Q99714	3‐hydroxyacyl‐CoA dehydrogenase type‐2	[HCD2_HUMAN]
WM	HCD	SKOR2	Q2VWA4	SKI family transcriptional corepressor 2	[SKOR2_HUMAN]

Note

The text in bold indicates proteins associated with calcium metabolism.

### 3.5 Functional Classification

Functional classification using QuickGO extracted 318 GO terms associated with the keyword calcium. The next stage was to determine which of the 1391 proteins identified in this investigation are associated with any of these 318 “calcium” GO terms. We found that 462 of our identified proteins to be associated with a total of 178 of the 318 calcium GO’s (shown in Data File S2).

From these 462 proteins, 201 (43%) could be matched to GNs, which is the output format of QuickGO.

Notably, FAM20A is linked to GO terms: calcium ion homeostasis (GO: 0055074) and enamel mineralization (GO: 0070166). From this list of 201, 21 proteins (1 from whole milk and 20 from MFGM fraction) were from our 136 proteins previously undiscovered in whole milk, Table 2.

**TABLE 2:** Previously undiscovered proteins in human milk associated with calcium metabolism

	Milk fraction	Accession #	Gene name	Description	GO ID	GO name	Aspect
1	MFGM	Q9UHX3	ADGRE2	Adhesion G protein‐coupled receptor	GO:0005509	Calcium ion binding	Function
2	MFGM	P78537	BLOC1S1	Biogenesis of lysosome‐related organelles complex 1 subunit	GO:0060155	Platelet dense granule organization	Process
3	MFGM	Q6QNY1	BLOC1S2	Biogenesis of lysosome‐related organelles complex 1 subunit 2	GO:0060155	Platelet dense granule organization	Process
4	MFGM	Q6QNY0	BLOC1S3	Biogenesis of lysosome‐related organelles complex 1 subunit 3	GO:0060155	Platelet dense granule organization	Process
5	MFGM	P10092	CALCB	Calcitonin gene‐related peptide	GO:0006874	Cellular calcium ion homeostasis	Process
6	MFGM	Q96EV8	DTNBP1	Dysbindin	GO:0060155	Platelet dense granule organization	Process
7	MFGM	P42566	EPS15	Epidermal growth factor receptor substrate 15	GO:0005509	Calcium ion binding	Function
8	MFGM	Q9UBC2	EPS15L1	Epidermal growth factor receptor substrate 15‐like 1	GO:0005509	Calcium ion binding	Function
9	MFGM	Q96HE7	ERO1A	ERO1‐like protein alpha	GO:0051209	Release of sequestered calcium ion into cytosol	Process
10	MFGM	P08709	F7	Coagulation factor VII	GO:0005509	Calcium ion binding	Function
11	MFGM	P00740	F9	Coagulation factor IX	GO:0005509	Calcium ion binding	Function
12	MFGM	Q9Y680	FKBP7	Peptidyl‐prolyl cis‐trans isomerase FKBP7	GO:0005509	Calcium ion binding	Function
13	Whole milk	P06241	FYN	Tyrosine‐protein kinase Fyn	GO:0006816	Calcium ion transport	Process
14	MFGM	Q16891	IMMT	MICOS complex subunit MIC60	GO:0051560	Mitochondrial calcium ion homeostasis	Process
15	MFGM	P17252	PRKCA	Protein kinase C alpha type	GO:0004698	Calcium‐dependent protein kinase C activity	Function
16	MFGM	P22891	PROZ	Vitamin K‐dependent protein Z	GO:0005509	Calcium ion binding	Function
17	MFGM	Q15042	RAB3GAP1	Rab3 GTPase‐activating protein catalytic subunit	GO:1903233	Regulation of calcium ion‐dependent exocytosis of neurotransmitter	Process
18	MFGM	Q14257	RCN2	Reticulocalbin−2	GO:0005509	Calcium ion binding	Function
19	MFGM	Q9UJZ1	STOML2	Stomatin‐like protein 2, mitochondrial	GO:0006851 GO:0006874	Mitochondrial calcium ion transport Cellular calcium ion homeostasis	Process
20	MFGM	Q9HCH5	SYTL2	Synaptotagmin‐like protein 2	GO:0005509 GO:0005544	Calcium ion binding calcium‐dependent phospholipid binding	Function
21	MFGM	P62760	VSNL1	Visinin‐like protein 1	GO:0005509	Calcium ion binding	Function

### 3.6 Network Analysis

**FIGURE 6:**
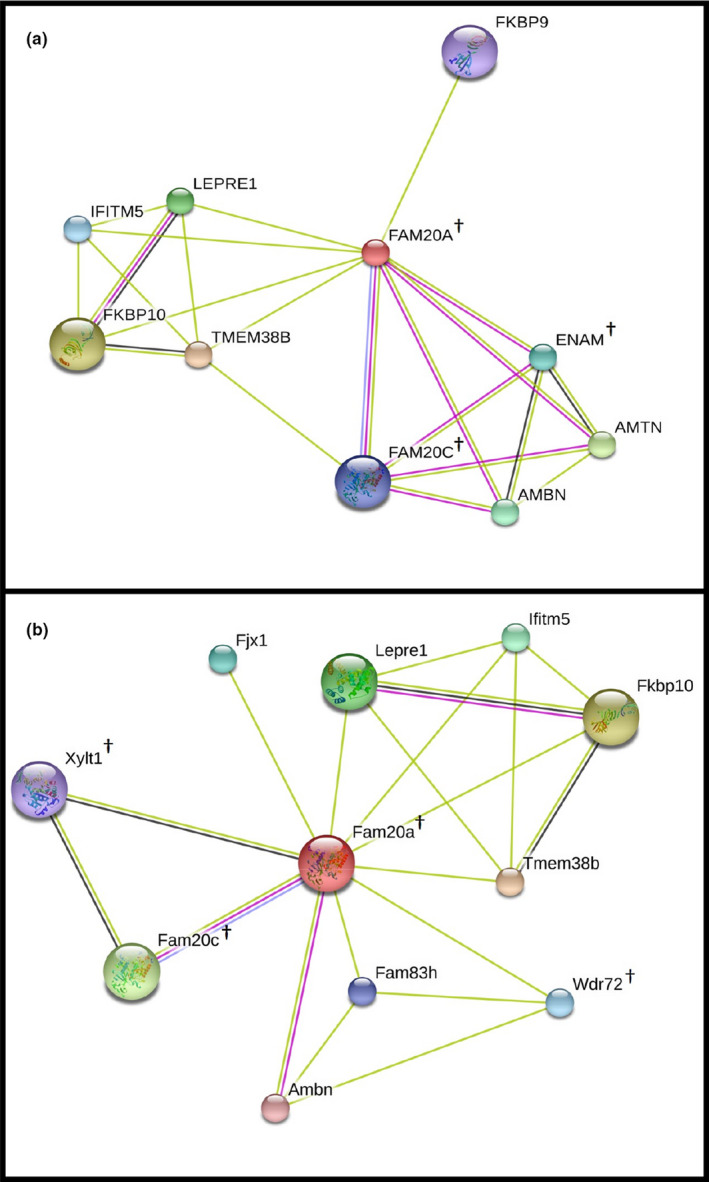
Interaction of known protein with FAM20A in (a) *Homo sapiens* and *Mus musculus* (b). STRING analysis reveals the interaction of FAM20A with nine and 10 other proteins in human and mouse, respectively. The relevant proteins are given as gene names and are shown as nodes. These proteins are produced by a single protein‐coding gene locus. The filled nodes symbolize 3D structure is either known or it is predicted. The edges (lines) represent either experimentally determined interaction (pink), or through text mining (green), or its co‐expression (black) and protein homology (purple). ^†^Proteins that were also identified in our dataset

Network analysis was performed using STRING to identify proteins that have a known interaction with FAM20A in humans (*H*. *sapiens*) or mice (*Mus musculus)* and consequently to see whether these proteins are present within our dataset and in the previously cataloged human milk proteome (Figure 6). This analysis revealed FAM20A interactions with nine and ten other proteins in *H*. *sapiens* and *M*. *musculus*, respectively. The GN, description and functions of these proteins are detailed in Table 3, we were only able to identify FAM20C, ENAM, XYLT1, and Wdr72 from our dataset and these proteins have been previously listed as part of the milk proteome (Lu et al., 2018; Ma et al., 2019; Yang et al., 2017; Zhu et al., 2019). We were unable to find any of these proteins in our list of newly discovered proteins in human milk.

**TABLE 3:** STRING analysis of proteins known to interact with FAM20A

Gene name		Description	Function
*Homo sapiens*	*Mus Musculus*		
**ENAM**	**No interaction**	Enamelin	Involved in mineralization of enamel
**AMTN**	**No interaction**	Amelotin	Promotes calcium phosphate mineralization
**FKBP9**	**No interaction**	Peptidyl‐prolyl cis‐trans isomerase FKBP9	Involved in protein folding
**AMBN**	**Ambn**	Ameloblastin	Involved in mineralization of enamel
**FAM20C**	**Fam20c**	Extracellular serine/threonine protein kinase	Phosphorylates proteins with a Ser‐x‐Glu/pSer motifs in the secretory pathway
**TMEM38B**	**Tmem38b**	Trimeric intracellular cation channel type B	Channel allowing rapid release of intracellular calcium
**FKB10**	**Fkb10**	Peptidyl‐prolyl cis‐trans isomerase FKBP1B	Involved in protein folding
LEPRE1 **(P3H1)**	Lepre **(P3h1)**	Prolyl 3‐hydroxylase 1	Involved in post translational modification of collagen
**IFITM5**	**Ifitm5**	Interferon‐induced transmembrane protein 5	Plays a role in bone mineralization
**No interaction**	**Fjx1**	Four‐jointed box protein 1	Descendants from the four‐jointed (FJ) family of protein kinases
**No interaction**	**Xylt1**	Xylosyltransferase 1	Involved in the synthesis of glycosaminoglycan
**No interaction**	**Wdr72**	WD repeat‐containing protein 72	Involved in mineralization of enamel
**No interaction**	**Fam83h**	Protein FAM83H	Involved in mineralization of enamel

Note

KEY: Bold = HUGO Gene Names and non‐bold = alternative Gene Names.

## 4 DISCUSSION

Calcium is essential for many cellular functions such as nerve impulse transmission, muscular contraction, hormone secretion, and intracellular adhesion and for bone mineralization. Intracellular calcium levels need to be tightly maintained; dysregulation can lead to severe cell dysfunction and death.

Ectopic calcification may also occur due to dysregulation of calcium homeostasis. Pathological calcification is associated with a number of medical conditions such as abdominal aortic aneurysm (Buijs et al., 2013) and chronic kidney disease. Tumor calcification is also common in breast cancer (Mischak et al., 2007).

Surprisingly, as milk is rich in minerals such as calcium and phosphates there seems not to be any evidence of calcification in the tissue during lactation. However, there are a few case reports of post‐lactation micro‐calcifications (Giron et al., 2004; Stucker et al., 2000). In these individual cases, there is a strong family history of breast cancer where calcifications have been noted in the breast tissue.

The processes that normally maintain calcium balance can be delineated by studying epithelial tissues where calcification is occurring. Since biopsies can be difficult to obtain from relevant cohorts this study has used human milk to identify and quantify FAM20A, a protein that is known to be involved in calcium regulation. The availability of samples in this investigation was limited and collected from specific lactating donors on an *ad hoc* basis. This resulted in three samples being collected for the detection and quantification of FAM20A.

Several studies have identified FAM20A in human milk through the use of mass spectrometry (Gao et al., 2012; Mange et al., 2013; Zhang et al., 2013). Here we performed the first comparative quantitative study using a targeted HCD MS/MS fragmentation experiment. This involved combining shotgun CID MS/MS and targeted HCD MS/MS fragmentation experiments to provide relative quantification across the three different milk samples. Furthermore, we performed proteomic analyses to establish potential functional network of proteins involved in calcium metabolism in milk and confirm whether any of these or existing proteins have any interaction with FAM20A as shown through the STRING analysis in Figure 6.

Despite low concentrations of FAM20A mRNA previously reported within the MFGM fraction of human milk (Lemay et al., 2013), we were unable to identify the protein within our pooled MFGM fraction.

Instead, using a shotgun CID MS/MS fragmentation experiment, the FAM20A protein, based on the combined peak area of the two peptides, was identified at low concentrations (1.6 µM) in human milk. This calculated amount is speculative, and a larger sample cohort would be needed to determine the concentration range in the human population. Regardless of the samples being handled differently, the difficulties of human milk storage conditions in neonatal hospitals are widely recognized. Additionally, an investigation on the impact of storage conditions on the breast milk peptidome for MS experiments demonstrated that the majority of peptides kept quite stable when stored at −80°C, −20°C, or even at 4°C (Howland et al., 2020).

Previous work by Nalbant et al. (2005), has shown FAM20A to be a secreted glycoprotein. Therefore, it is likely the FAM20A detected in our whole milk is expressed within the lactocytes and secreted into the whey fraction of human milk. This concurs with previous findings (Beck et al., 2015; Gao et al., 2012; Mange et al., 2013; Zhang et al., 2016), identifying FAM20A in the whey fraction of human milk. The FAM20A mRNA detected by Lemay et al. (2013) may be encapsulated within the MFGM during its development. The development starts with the formation of lipids within the endoplasmic reticulum (ER). Secretion of this lipid occurs by it being enveloped by the plasma membrane at the apical side of the lactocytes, taking with it the mRNA as well as protein remnants from these cells.

Subsequent analysis involved HCD MS/MS fragmentation experiment to quantify all proteins including FAM20A in whole milk from the three individuals with and without PI. This procedure quantified 247 proteins and FAM20A protein was not identified in this list. This suggests that without a hypothesis‐driven question these quantitative platforms can miss out proteins of clinical relevance. It was only through a targeted HCD MS/MS experiment that we were then able to successfully quantify FAM20A up to 44 weeks milk postpartum.

Little is known regarding the role of FAM20A, though there is an association that FAM20A prevents calcification. Furthermore, there is evidence suggesting that FAM20A, a pseudokinase, forms a functional unit with FAM20C and this complex enhances the ability to phosphorylate several proteins with serine‐x‐glutamic acid/phosphoserine motifs. One of these proteins is the secreted phosphoprotein 1 (SPP1) (Cui et al., 2015; Ohyama et al., 2016) also known as Osteopontin (OPN). This protein was identified in both the whole milk and MFGM fraction.

However, network analysis with STRING (as accessed in May 2017) did not confirm an association of SPP1 with FAM20A; we know STRING is an evolving application that employs algorithms based on known knowledge and prediction of protein to protein interaction and therefore not surprising at the time of analysis SPP1 was not known in the database. Furthermore, despite STRING being a currently accepted application for protein networks, any interactions identified would need further experimental validation to confirm any putative biological interactions.

STRING did identify direct interaction (as indicated by selecting the first shelf in the settings) with 13 other proteins with FAM20A (Table 3). From these FAM20C, ENAM, XYLT1, and WDR72 have previously been identified in human milk. As already mentioned, FAM20C forms a functional unit with FAM20A and enhances phosphorylation of secreted proteins such as SPP1, RCN1, and NUCB1 (Cui et al., 2015; Ohyama et al., 2016). Furthermore mutations in FAM20C have been associated with lethal bone dysplasia (Raine syndrome); a condition characterized by over‐calcification of bone and other non‐mineralized tissues (Ababneh et al., 2013; Acevedo et al., 2015; Fradin et al., 2011; Seidahmed et al., 2015; Simpson et al., 2007, 2009) Interestingly, mutations in WDR72 (El‐Sayed et al., 2009) and ENAM (Rajpar et al., 2001) are also causative for amelogenesis imperfecta, emphasizing the essential role of these proteins in normal physiological calcification processes, as disruptions in these genes lead to loss of, thinning or softening of enamel as aberration (Slootweg, 2007). For example, loss of WDR72 results in mislocalization of the sodium calcium potassium exchanger 4 (SLC24A4) away from the distal membrane and into the cytosol of enamel‐depositing ameloblast cells (Wang et al., 2015). WDR72 has also been identified as causative for distal renal tubular acidosis, which in turn is associated with nephrocalcinosis and thus shares important parallels with FAM20A (Zhang et al., 2019). These ameloblast cells, which are only present during tooth development, also secrete the enamel matrix protein, ENAM which is required for proper formation of enamel (Rajpar et al., 2001). Whereas point mutations in the XYLT1 and XYLT2, both of which code for a protein required for initiation of biosynthesis of proteoglycan in articulate cartilage, have been associated with decreased enzyme activity and early onset osteoarthritis (Schön et al., 2006).

Interestingly, calcification has not been reported in lactating breast tissue of ERS patients with recessive mutations in *FAM20A*. This could be a result of the extreme rarity of this disorder or be compatible with the presence of other proteins related to calcium metabolism.

QuickGo and STRING applications are constantly undergoing changes due to ever‐increasing knowledge of protein multi‐location and functional moonlighting. Functional classification analysis only revealed a 43% (201 proteins) match to our original input; a disparity likely the result of variations in the output generated by two unsynchronized applications (QuickGo and Proteome Discoverer). Furthermore, 21/201 proteins are from our list of previously undiscovered proteins in human milk. Interestingly one of these is the calcitonin gene‐related peptide‐2 CALCB (GO: 0006874, cellular calcium ion homeostasis) which was identified in the MFGM fraction. Its family member, the hormone calcitonin is involved in regulating calcium levels (Steenbergh et al., 1985) and it is likely CACLB could also be involved in maintaining Ca^2+^ levels. So far studies have shown that CALCB is expressed in the enteric and stomach neuronal system and plays an important role (1) as a vasodilator in stimulating milk production (Smillie & Brain, 2011; Thulesen et al., 1994) and (2) in maintaining the mucosal integrity and gastric acid regulation (Beglinger et al., 1988; Feng et al., 2011; Lenz et al., 1985; Ohno et al., 2008; Pappas et al., 1986; Tache, 1992). In addition supplementation of CALCB has been shown to relieve gastric hyperacidity (Beglinger et al., 1988).

Human milk has recently been shown to activate the coagulations system by triggering the coagulation tissue factor (TF) located in extracellular vesicle (Hu et al., 2020). Additionally, from our unique list of calcium metabolism proteins, F7 (Broze, 2001), F9 (Millar et al., 2000) and PROZ (Davie & Fujikawa, 1975) are involved in blood coagulation. F7 is a high‐affinity receptor and a co‐factor for F7. Upon vascular injury, F7 contacts TF, and is cleaved to its active form; this reaction is the primary event in blood coagulation. The complex of TF and activated factor VII serves to activate other coagulation factors including F9 (Millar et al., 2000). Furthermore, the BLOC1 complex is also known to regulate coagulation (Merideth et al., 2020), and its members BLOC1S1, BLOC1S2, BLOC1S3^, (^Starcevic & Dell'Angelica, 2004) and DTNBP3 (Nazarian et al., 2006) are part of our newly discovered list of proteins in human milk that are associated with calcium metabolism.

### 4.1 Limitations

The principal limitation of our study is the samples were collected at different time points. Furthermore, due to the inherent limitation of labeling kits for quantitative proteomics the maximum number of samples that can be analyzed is 16. Hence there are no quantitative proteomics studies using large numbers of human milk samples, probably reflecting the difficulties in obtaining such a collection. We therefore can only comment on the presence of FAM20A in human milk but not on how the concentration may vary (1) between individuals and (2) the number of weeks post‐partum. Nevertheless, the identification of FAM20A in all samples supports a role for this protein in preventing the calcification of epithelial tissues. Further studies using the sample from defined lactation time points would be needed to better understand this.

## 5 CONCLUSION

In this current study, we were able to quantify FAM20A in transitional and mature human milk via the MS/MS driven proteomics targeted approach. The different fragmentation protocols used in this investigation show variable results in terms of type and number of proteins identified. Thus, the study highlights the importance of combining both traditional shotgun CID MS/MS fragmentation experiments with targeted HCD MS/MS fragmentation experiment for the analysis of low abundance proteins in hypothesis‐driven research.

Additionally, the 136 newly discovered proteins are now cataloged to the evolving human milk proteome. Interestingly, 21 of the newly discovered proteins associated with calcium regulation, 11 are associated with calcium‐binding ion (GO: 0005509), and 2 were associated with cellular calcium homeostasis (GO: 0006874). We speculate that these proteins could potentially be key players in buffering the large amount of calcium passing through MEC, hence preventing calcification of the secretory epithelial tissue of breast during lactation.

## CONFLICT OF INTEREST

There is no conflict of interest.

## AUTHOR CONTRIBUTIONS

VP wrote the manuscript, prepared the samples, and analyzed the data. EK was involved in analysis of the data. GW contributed to sample preparation and optimizing mass spectrometry protocols. DB obtained funding and revised the manuscript. KS, SBW, MB, and NH contributed in writing of the manuscript. GJ and NI contributed to the design of the study. JGZ revised the manuscript and contributed to the data analysis. RK obtained funding and contributed to the conception of this study and was involved in the overall design of the study, JW contributed to the overall design of the study and performed all mass spectrometry experiments.

## ETHICAL APPROVAL

This work has been performed in accordance with the Declaration of Helsinki under a protocol approved by the NHS research ethics committee (05/Q0508/6).

## Supporting information



Data File S1

Data File S2

Data File S1‐S2
