# Supplementary material for: Quantification of FAM20A in human milk and identification of calcium metabolism proteins
Source: Physiol Rep. 2021 Dec 27;9(24):e15150. doi: 10.14814/phy2.15150 (PMC8711012; doi:10.14814/phy2.15150)
Supplement: Supplementary file 3 — Data File S1‐S2 [file PHY2-9-e15150-s003.docx]

**Supplementary information**

**Data file S1. Proteomic profiling of human milk**

Proteins identified in human milk and MFGM fraction with the different fragmentation mass spectrometer strategies together with the entire milk proteome to date.

**Data file S2.** **Proteins associated with calcium regulation**

Proteins involved in calcium regulation from the entire human milk proteome including those newly identified in this investigation.
